# Exercise echocardiography for the assessment of pulmonary hypertension in systemic sclerosis: a systematic review

**DOI:** 10.1186/s13075-016-1051-9

**Published:** 2016-07-02

**Authors:** Rui Baptista, Sara Serra, Rui Martins, Rogério Teixeira, Graça Castro, Maria João Salvador, José António Pereira da Silva, Lèlita Santos, Pedro Monteiro, Mariano Pêgo

**Affiliations:** Department of Cardiology, Centro Hospitalar e Universitário de Coimbra, Praceta Mota Pinto, 3000-001 Coimbra, Portugal; Faculty of Medicine, University of Coimbra, Coimbra, Portugal; Department of Rheumatology, Centro Hospitalar e Universitário de Coimbra, Coimbra, Portugal; Department of Internal Medicine, Centro Hospitalar e Universitário de Coimbra, Coimbra, Portugal

**Keywords:** Exercise, Echocardiography, Systemic sclerosis, Pulmonary hypertension, Scleroderma

## Abstract

**Background:**

Pulmonary arterial hypertension (PAH) complicates the course of systemic sclerosis (SSc) and is associated with poor prognosis. The elevation of systolic pulmonary arterial pressure (sPAP) during exercise in patients with SSc with normal resting haemodynamics may anticipate the development of PAH. Exercise echocardiography (ExEcho) has been proposed as a useful technique to identify exercise-induced increases in sPAP, but it is unclear how to clinically interpret these findings. In this systematic review, we summarize the available evidence on the role of exercise echocardiography to estimate exercise-induced elevations in pulmonary and left heart filling pressures in patients with systemic sclerosis.

**Methods:**

We conducted a systematic review of the literature using MEDLINE, Cochrane Library and Web of Knowledge, using the vocabulary terms: (‘systemic sclerosis’ OR ‘scleroderma’) AND (‘exercise echocardiography’) AND (‘pulmonary hypertension’). Studies including patients with SSc without a prior diagnosis of PAH, and subjected to exercise echocardiography were included. All searches were limited to English and were augmented by review of bibliographic references from the included studies. The quality of evidence was assessed by the Effective Public Health Practice Project system.

**Results:**

We identified 15 studies enrolling 1242 patients, who were mostly middle-aged and female. Several exercise methods were used (cycloergometer, treadmill and Master’s two step), with different protocols and positions (supine, semi-supine, upright); definition of a positive test also varied widely. Resting estimated sPAP levels varied from 18 to 35 mm Hg, all in the normal range. The weighted means for estimated sPAP were 22.2 ± 2.9 mmHg at rest and 43.0 ± 4.3 mmHg on exercise; more than half of the studies reported mean exercise sPAP ≥40 mmHg. The assessment of left ventricular diastolic function on peak exercise was reported in a minority of studies; however, when assessed, surrogate variables of left ventricular (LV) diastolic dysfunction were associated with higher sPAP on exercise.

**Conclusions:**

We found very high heterogeneity in the methods, the protocols and the estimated sPAP response to exercise. LV diastolic dysfunction was common and was associated with greater elevation of sPAP on exercise.

**Electronic supplementary material:**

The online version of this article (doi:10.1186/s13075-016-1051-9) contains supplementary material, which is available to authorized users.

## Background

Systemic sclerosis (SSc) is an autoimmune disorder characterised by autoantibody production, microvascular lesions and collagen deposition [1] and can be complicated by pulmonary arterial hypertension (PAH) in 8 to 20 % of patients [2]. PAH remodelling affects the small pulmonary arteries, leading to progressive increase in pulmonary vascular resistance (PVR) and right heart failure [3]. Importantly, histopathological evidence of pulmonary arteriopathy has been reported in up to 72 % of patients with SSc, raising the possibility that subclinical features are present well before the PAH diagnosis [4]. Therefore, early diagnosis is of importance, not only because of the rapid progression of the disease but also because it is critical to initiate early treatment [5].

Echocardiography is a useful tool for pulmonary hypertension (PH) screening, as it estimates systolic PAP (sPAP), assesses right ventricular remodelling, identifies morphological abnormalities that can indicate the aetiology and may be used to evaluate treatment effectiveness [6]. However, a resting echocardiogram has limited accuracy to diagnose elevations in pulmonary pressures in SSc [7–9]. Stress tests performed during exercise have been used increasingly in cardiology to better characterise haemodynamic changes and therefore, a strong rationale exists to suggest that reduced pulmonary vascular reserve may signal a subclinical phase of pulmonary vascular disease (PVD) [10]. The definition of exercise-induced PH (EIPH) has been present in former PH guidelines, being defined by a mean PAP (mPAP) >30 mmHg. However, it has been abandoned due to lack of standardisation, prognostic impact assessment and overlap with normal subjects [11]. Nonetheless, performing an exercise echocardiogram may be an advantageous approach, as it allows not only estimation of exercise-induced changes in sPAP but also quantification of changes in cardiac output (CO) and left ventricular (LV) diastolic filling pressures, two determinants of variation in pulmonary pressure. Some authors suggest that this early exercise PAH phase is more amenable to treatment; others suggest it may be stable with no pathological implications [12, 13].

In this systematic review, we summarize the available evidence on the role of exercise echocardiography to estimate exercise-induced elevations in pulmonary pressures and in left heart filling pressures in patients with SSc without resting PH.

## Methods

The methods used conformed to the Meta-analysis of Observational Studies in Epidemiology [14] and the Cochrane Collaboration [15] recommendations.

### Selection criteria and search strategy

The literature search was conducted between January 2013 and December 2015 and comprised peer-reviewed original research that investigated estimated EIPH as a primary endpoint in patients with SSc using exercise echocardiography. The search resources included MEDLINE, Cochrane Library and Web of Knowledge, using the search terms: (‘systemic sclerosis’ OR ‘scleroderma’) AND (‘exercise echocardiography’) AND (‘pulmonary hypertension’). Studies that were already known to the authors of this review (based on previous work or familiarity with the research area) were also included in the review. The search was limited to English language articles published from 1995 onwards. Publications reporting no original data or without a clear description of the research methods were excluded. Studies that did not present estimated sPAP results for patients with SSc were excluded. Conference abstracts or results posted in trial registries were excluded. The grey literature was not searched.

### Data extraction and assessment

**Fig. 1:**
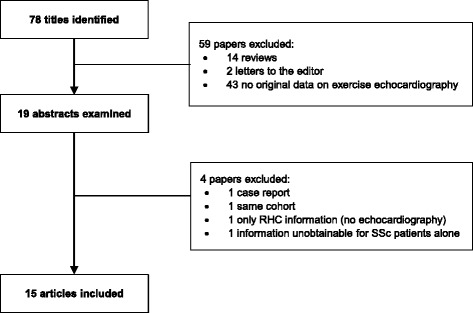
Search strategy and exclusion process for studies on exercise echocardiography in patients with systemic sclerosis (*SSc*). RHC: right heart catheterization

Study selection was performed by the investigators RB (cardiologist) and SS (rheumatologist). References were managed using Mendeley Desktop software (V.1.12.3). We contacted the authors who reported sPAP by groups of patients defined by a cutoff for exercise-induced sPAP, but not for overall patients. Retrieved papers were hand-searched for additional references. Details of the literature search process are outlined in the flow chart (Fig. 1). Eligible studies included adult (18 years old or more) patients with SSc (either diffuse or limited), subjected to a stress protocol with exercise echocardiography. Patients were enrolled consecutively in all but one study, where only patients at high risk of developing PAH were included. As the main goal of exercise echocardiography in SSc is to identify patients with pre-capillary (PAH) elevations in pulmonary pressures, most authors aimed to exclude patients at high risk of group-2 PH, as those with uncontrolled systemic hypertension or a history or evidence of significant cardiovascular or lung disease (including coronary artery disease, LV hypertrophy, myocardial ischaemia and severe valvular conditions). A prior PAH diagnosis was also a criterion for study exclusion.

All studies had a similar design. Patients were submitted to echocardiography at rest (before exercise) and during or immediately (within the first minute) after exercise. The following variables were extracted when available: year of publication, sample size, gender ratio, age, lung function test parameters (forced vital capacity (FVC), forced expiratory volume in the first second (FEV_1_) and total lung capacity (TLC)), carbon monoxide diffusion capacity (DL_CO_), type and date of SSc diagnosis, exercise method, time of exercise-induced estimated sPAP measurement, maximum achieved workload in metabolic equivalent of task (MET), patient position (supine or upright), feasibility and intra and inter-observer variability (the latter two parameters are presented in Additional file 1). Cardiovascular variables collected included estimated resting right atrial pressure (RAP), tricuspid regurgitation-derived estimated sPAP (at rest and peak exercise), heart rate (HR) (at rest and peak exercise), estimated CO and cardiac index (CI) (at rest and peak exercise) and estimated pulmonary vascular resistance (PVR). In addition, diastolic dysfunction markers were retrieved when available, namely the ratio of early diastolic (E) and late diastolic (A) transvalvular velocities (E/A) for each ventricle and the ratio of early diastolic (E) and tissue Doppler-derived early (e’) and atrial (a’) diastolic mitral annular velocity to early diastolic (E) wave (E/e’). All data retrieved from the studies were relevant to the population characteristics, study design, type of exercise endured and outcomes of interest. In the scope of this review, the weighted mean and standard deviation for estimated sPAP (both at rest and peak exercise) was also calculated.

### Grading the quality of evidence of included studies

The Effective Public Health Practice Project (EPHPP) was used by two of the authors (RB and SS) to rate the quality of the evidence in the reviewed studies (Additional file 2) [16]. Each study was assigned a score category of strong, moderate or weak. Studies were graded by independent reviews; when the original ratings disagreed, they underwent a resolution review consensus. Double entry of data was performed for three studies (20 % of studies) and demonstrated a high level of accuracy (98.7 %).

## Results

A total of 78 publications were identified in the literature search. Of these, 19 were retrieved and analysed for eligibility. A total of 15 studies were eligible for inclusion in the review, and these were published between 1996 and 2015 (Fig. 1) [8, 17–30]. The characteristics of each eligible study are presented in Table 1.

**Table 1:** Main characteristics of studies and patients

First author	Publication year	Condition	Sample size	Female gender (%)	Age (years)	Mean time since diagnosis	Enrolment criteria
Mininni [25]	1996	SSc	9	78 %	56	27 m	Consecutive
Alkotob [17]	2006	SSc	65	86 %	51	---	Consecutive
Collins^a^[21]	2006	DSSc	9	100 %	59	---	Consecutive
Collins^b^[21]	2006	LSSc	10	100 %	52	---	
Pignone [26]	2007	LSSc	27	89 %	50	7 y	Consecutive
Huez [24]	2007	SSc	8	92 %	54	16 m	Consecutive
Callejas-Rubio [29]	2008	SSc	41	--	53	9 y	Consecutive
Steen [27]	2008	SSc	54	94 %	53	---	At high risk of PH^d^
Reichenberger [8]	2009	SSc	33	94 %	54	9 y	Consecutive
D’Alto [22]	2010	SSc	172	90 %	52	---	Consecutive
Ciurzynski [20]	2011	SSc	67	96 %	57	---	Consecutive
Baptista [18]	2013	SSc	23	96 %	58	---	Consecutive
Gargani [23]	2013	SSc	164	91 %	58	11 y	Consecutive
Voilliot [29]	2014	SSc	45	76 %	54	---	Consecutive
Suzuki [28]	2014	SSc	494	89 %	56	---	Consecutive
Nagel^c^[30]	2015	SSc	21	84 %	58	12 y	Consecutive

Quantitative variables (age) reported as means. Female and male patients represented by counts. ^a^Results for patients with diffuse systemic sclerosis (DSSc). ^b^Rresults for patients with limited systemic sclerosis (LSSc). ^c^Results are for the full population studied (including patients unaware of having pulmonary hypertension). ^d^Dyspnea on exertion, carbon monoxide diffusion capacity (DL_CO_) <60 % of predicted, forced vital capacity (FVC) <60 % of predicted, FVC %/DLCO % >1.6, or resting right ventricular systolic pressure on echocardiogram >30 mmHg but <50 mmHg. *m* months, *SSc* systemic sclerosis, *y* years

### Demographic variables

The demographic characteristics are presented in Table 1. A total of 1242 patients was assessed, with a large female majority (range 76–100 %). The mean age in each study ranged from 50–58 years and the mean time since SSc diagnosis varied from 16 months to 12 years.

### Stress protocols and measurements

Exercise echocardiography protocols and maximum workload levels are described in Additional file 3. Of the selected studies, 10 used a cycloergometer in the supine or semi-supine position. Half of the studies estimated exercise-induced sPAP immediately after or within one to two minutes after exercise. HR was measured in 10 of the selected studies. At rest, mean HR ranged between 71 and 82 bpm. Mean peak exercise HR varied between 115 and 150 bpm, signalling for moderate intensity exercise, given the mean age of the population (54 years) and the expected maximum HR for this age group (approximately 166 bpm).

**Fig. 2:**
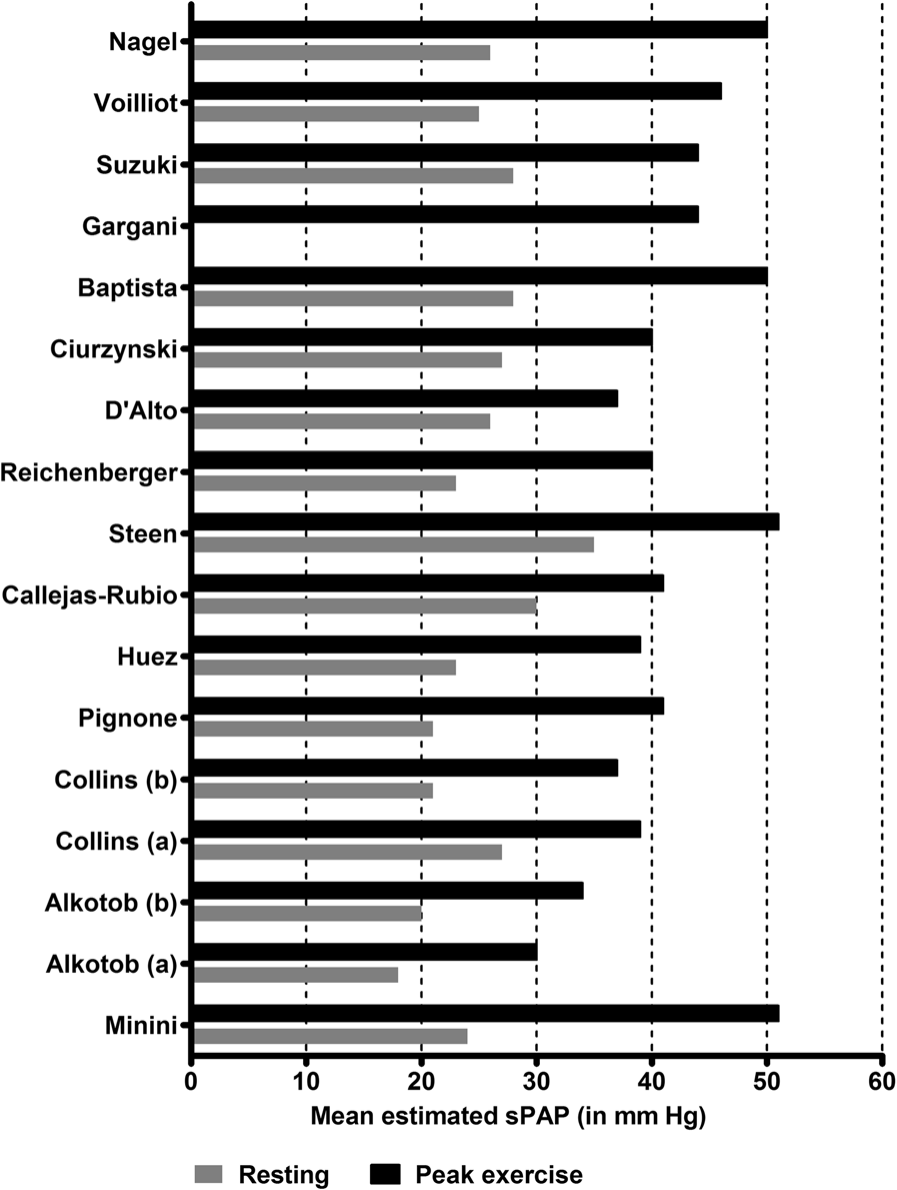
Mean pulmonary arterial systolic pressure by study. Results from Alkotob et al. are divided into *(a)* patients with pulmonary fibrosis and *(b)* patients without pulmonary fibrosis. Results from Collins *et al* are divided into *(a)* patients with diffuse systemic sclerosis and *(b)* patients with limited systemic sclerosis. The weighted mean for estimated systolic pulmonary arterial pressure (*sPAP*) was 22.2 ± 2.9 mmHg at rest and 43.0 ± 4.3 mmHg at peak exercise

**Fig. 3:**
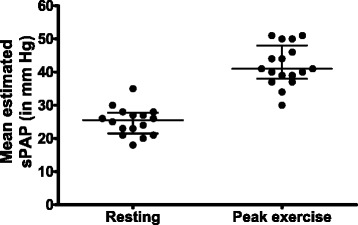
Median systolic pulmonary arterial pressure (*sPAP*) (with interquartile range) estimated by exercise echocardiography in resting and peak exercise conditions for each study

Mean estimated sPAP is shown by study in Fig. 2. Resting sPAP varied from 18–35 mmHg, all in the normal range. At peak exercise, mean sPAP ranged from 30–51 mmHg, with half of the studies reporting a mean exercise sPAP ≥40 mmHg (Fig. 3). The weighted means for estimated sPAP were 22.2 ± 2.9 mmHg at rest and 43.0 ± 4.3 mmHg at peak exercise. Most of the studies defined a minimum cutoff for EIPH that would define a clinically relevant hypertensive response (a “positive test”) for the exercise echocardiographic study. These thresholds are shown in Additional file 4; the proportion of positive tests ranged from 12–67 %.

Patients who exercised in an upright position on a treadmill had numerically smaller elevation in sPAP than patients assessed on a cycloergometer in a semi-supine position (40 ± 7 vs. 47 ± 4 mmHg) (Additional file 5). However, this might be due to the fact that upright echocardiographic assessments of patients on the treadmill were always performed immediately after exercise. In the studies where measurements were taken at peak exercise, mean sPAP was 45 ± 5 mmHg, whereas in studies were it was collected immediately after exercise was 40 ± 3 mmHg.

### Left ventricular diastolic dysfunction markers

As SSc commonly affects the LV, the elevation of sPAP during exercise might also be due to backward transmission of elevated LV end-diastolic pressure (LVEDP). Therefore, the assessment of a surrogate of LVEDP is critical during the exercise test. Table 2 shows the results from eight studies that reported these markers among patients with and without positive tests in the exercise echocardiogram in resting conditions (described in Additional file 5 for each study). Most studies report signs of resting LV diastolic dysfunction in the group of patients who exhibited EIPH. Only three studies reported LV E/e’ during peak exercise; all demonstrated a higher exercise E/e’ in the patients with a positive exercise test.

**Table 2:** Echocardiographic diastolic dysfunction markers

		First author																
Condition		Alkotob (n = 65)	Pignone (n = 27)			Huez (n = 25)	D’Alto (n = 172)	Baptista (n = 23)			Gargani (n = 164)		Voilliot (n = 45)			Suzuki (n = 494)		
		Total	Total	≤40 mmHg	>40 mmHg	Total	Total	Total	<50 mHg	≥50 mmHg	<50 mmHg	≥50 mmHg	Total	≤50 mmHg	>50 mmHg	Total	<50 mmHg	≥50 mmHg
Rest	RV E/A						0.9				1.0*	0.7*						
	LV E/A	1.2	1.1	1.2	1.0	1.3	1.2	1.1	1.1	1.1			1.1	1.1	1.1	1.2	1.2*	1.1*
	E/e’							10.2	9.9	10.5	6.6*	7.5*	6.0	5.5*	6.8*	9.6	9.2*	10.7*
Exercise	RV E/A						0.7											
	LV E/A							1.2	1.3	1.1			1.1	1.1	1.0			
	E/e’							10.0	9.4	10.5			6.8	5.7*	9.2*	10.7	10.3*	11.8*

Results presented as means. *Comparison between patients with maximum exercise-induced systolic pulmonary arterial pressure (sPAP) <50 mmHg and ≥50 mmHg statistically significantly different (*p* < 0.05). *E/A* ratio of early diastolic (E) and late diastolic (A) transvalvular velocities, *E/e’* ratio of early diastolic (E) and early diastolic mitral annular velocity (e’), *LV* left ventricle, *RV* right ventricle

### Cardiac output, cardiac index and pulmonary vascular resistance

Table 3 shows the studies that evaluated CO, CI and PVR at rest and during/after exercise, with some performing comparisons between patients with and without a positive test. Baptista et al. [18] observed higher mean resting CO in patients with positive tests. Suzuki et al. and Voilliot et al. [28, 29] also observed higher mean PVR in patients with positive tests, both for resting and peak exercise PVR. Gargani et al. [23] also observed a difference, but for peak exercise PVR only and they also measured the ratio between changes in mean PAP (mPAP) and changes in CO (mPAP/CO) induced by exercise. Patients with positive tests had higher ratios compared with the remaining patients (*p* < 0.05). D’Alto et al. [22] also identified higher ΔsPAP/ΔCI ratios in patients with SSc, compared with controls; Voilliot et al. [29] demonstrated positive correlation between PVR and exercise sPAP.

**Table 3:** Echocardiographic-derived cardiac output, cardiac index and pulmonary vascular resistance

First author	Resting CO (L.min^1^)	Exercise CO (L.min^1^)	Resting CI (L.min^1^.m^2^)	Exercise CI (L.min^1^.m^2^)	Resting PVR (WU)	Exercise PVR (WU)
Huez						
Total	3.0	8.7				2.9
D’Alto						
Total			3.0	5.8		
Baptista						
Total	3.6	9.2				
sPAP <50 mmHg	3.8*	8.5				
sPAP ≥50 mmHg	5.1*	9.9				
Suzuki						
Total	5.5	7.6			1.7	1.9
sPAP <50 mmHg	5.5	7.6			1.6*	1.8*
sPAP ≥50 mmHg	5.5	7.6			1.9*	2.3*
Voilliot						
Total	3.7	7.2			1.9	2.5
sPAP ≥50 mmHg	3.9	7.7			1.4*	2.1*
sPAP >50 mmHg	3.5	6.5			2.6*	3.6*
Gargani						
sPAP <50 mmHg			2.5	4.6	1.7	2.0*
sPAP ≥50 mmHg			2.7	4.9	1.7	2.3*

Results are presented as means. *Comparison between patients with maximum exercise-induced systolic pulmonary arterial pressure (sPAP) <50 mmHg and ≥50 mmHg statistically significantly different (*p* < 0.05). *CI* cardiac index, *CO* cardiac output, *PVR* pulmonary vascular resistance, *WU* Wood units

### Lung function tests

DL_CO_ and spirometry measures are presented in Additional file 5. On evaluating the association between these parameters and sPAP, Steen et al. [27] reported that patients with DL_CO_ <50 % or with FVC %/DL_CO_ ratio >1.6 were more likely to have an increase in sPAP ≥20 mmHg at peak exercise (*p* < 0.001). Callejas-Rubio et al. [19] also observed negative correlation between exercise sPAP and DL_CO_.

## Discussion

We conducted a systematic review of the scientific literature assessing estimated sPAP by echocardiography during exercise in patients with SSc. The results demonstrate very high heterogeneity in (1) the methodology used to exercise the patients and to estimate pressures, (2) the definition of what is a positive response and (3) the assessment of the LV diastolic performance at rest and during exercise. Further, robust outcome data from patients with EIPH are also lacking, making it difficult to establish prognostic correlation.

### Methodology of the test

Several exercise methods, positions and protocols were used among the studies. It is well-known that the cardiopulmonary circulatory responses are different if patients exercise in the supine or upright position and may relate differently to symptoms; also, Doppler-derived parameters can change with position and are challenging to assess in the upright position [31–33]. However, we observed several methods (cycloergometer, Master’s two step and treadmill) and several positions (upright, supine and semi-supine) used for exercise. Moreover, the different stress protocols did not specify a predefined level of exertion to be achieved to define a test as being positive, as in other clinical scenarios such as coronary ischaemic disease, in a classical treadmill test. In combination, these factors may have accounted for the significant variability found in the workload and peak exercise heart rate among the different studies, and consequently, in the increased cardiac work necessary to elicit a significant haemodynamic response. Besides different workloads, Doppler-derived parameters can change with position. For instance, when a patient moves from the supine to the upright position, the E/e’ ratio can change, as both parameters are preload-dependent [34]. Additionally, correct Doppler assessment of the tricuspid regurgitant velocity needs correct alignment of the Doppler beam, something that can be difficult to achieve in a patient exercising upright on a treadmill. Therefore, the most reproducible approach is probably semi-supine exercise on a cycloergometer with a ramped stress protocol, as recently proposed [35].

Another important aspect is the timing of imaging acquisition. In most, but not all studies, estimated pressures were collected during and at peak exercise, while the patient was still exercising. However, some authors assessed the patient one or two minutes after the end of exercise. CO and heart rate pressure rapidly return to normal after cessation of exertion (due to reversal of splanchnic vasoconstriction and decreased venous return), and these findings may underestimate sPAP elevation (or the severity of diastolic dysfunction) [36]. In comparison with the other studies, the low proportion of patients with a positive test reported by Ciurzynski et al. may be due to the fact that sPAP was not measured during but after exercise [31–33]. Using a ramp protocol with a cycloergometer, the load can be maintained constant over approximately three minutes (at a pedalling rate of 55–65 bpm), enabling imaging acquisition during peak exercise (or when symptoms develop) instead of post-exercise measurements [35].

### Interpretation of an exercise echocardiography test

Importantly, exercise sPAP cannot be interpreted without information on two other determinants of pressure: flow and LVEDP [37]. Pulmonary flow was reported in six studies. In comparison to baseline CO, peak exercise CO ranged between twofold and threefold the baseline value, a small increase taking into consideration the expected increase in CO in healthy subjects (fourfold to tenfold) [38]. This may signify either (1) a limited capacity in patients with SSc to increase the CO during exercise or (2) an inability of patients with SSc to exercise to a higher workload due to other mechanisms such as lung fibrosis, osteoarticular issues, anaemia or deconditioning.

In general, the mean increase in sPAP across all studies was 15 mmHg, to a weighted mean level of 43 mmHg; most studies reported a value >40 mmHg. This elevation in pressure is probably higher than would be expected from the concomitant elevation in CO in this group of patients with SSc, taking into consideration that in normal individuals, exercise leads to smaller increases in sPAP (mean 34.3 ± 7.5 mmHg) under large elevations of CO (approximately 20 L.min^-1^) [38]. The importance of taking CO into consideration when reporting exercise haemodynamics is of paramount importance. In a study by Argiento et al., 19 of 25 normal individuals had an elevation of estimated sPAP >40 mmHg. However, the mean CO at peak exercise was 18.0 ± 4.2 L.min^-1^ [39], much higher than the values achieved by the patients with SSc. Therefore, for a CO <8–10 L.min^-1^ (similar to the ones reported in the SSc studies we reviewed), mPAP should be <30 mmHg (corresponding to an sPAP of approximately 47 mmHg), a threshold surpassed in the majority of the studies performed in patients with SSc.

LV diastolic dysfunction is a critical determinant of EIPH. As the LVEDP can contribute with more than 75 % to sPAP during exercise in patients with resting PH due to LV diastolic dysfunction [40], any analysis of exercise sPAP without concomitant assessment of a surrogate of LVEDP is difficult to interpret [41]. The studies that analysed resting and exercise LV diastolic parameters mostly used E/e’ as a marker of diastolic function [42]. Although arguably useful in the resting estimation of LV filling pressures, the use of E/e’ in exercise protocols is debatable and used in isolation, E/e’ is probably not sufficient to form conclusions about exercise-induced diastolic dysfunction [35, 43]. However, taking into consideration those limitations, most studies found that higher elevations of sPAP were associated with higher levels of either resting or peak E/e’, and many conclude that there is an association between impaired relaxation of the LV and an increase in sPAP, both at rest and on peak exercise. Therefore, one cannot diagnose the presence of pulmonary vascular disease based only on sPAP elevation upon exercise, as diastolic dysfunction is very prevalent in patients with SSc (at least when assessed using E/e’). In the future, multi-parametric algorithms may enable a more accurate diagnosis of diastolic dysfunction [35], namely, not only based on a single parameter, but also including clinical factors (such as the presence of hypertension, atrial fibrillation, diabetes or obesity), electrocardiographic data and echocardiographic markers of long-standing elevated LV filling pressures (such as LA area or the presence of LV hypertrophy) [44].

Due to the concurrent need for assessing CO along with pulmonary pressures and the limitations of establishing a diagnosis with only one sPAP value collected at some time point, a better method for evaluating the pressure-flow relationship in the pulmonary circulation might be by the analysis of ΔPAP/ΔCO slopes (i.e., total pulmonary resistance, TPR), calculated by the measurement of several pressure-flow points throughout the exercise period [10, 45]. An abnormal response to exercise is signalled by an mPAP >30 mmHg with an mPAP/CO slope >3 mmHg.L.^-1^.min^-1^ [3]. Steeper slopes were found in patients with SSc compared with controls [46]. Importantly, this calculation does not take in consideration LVEDP; therefore, it is unable to differentiate pre-capillary from post-capillary EIPH.

### Definition of a positive result on exercise echocardiography

The invasive right heart catheterization-based definition of EIPH (mPAP >30 mmHg) was abandoned in 2008 due to limited supporting data [11]. Similarly, for echo-derived sPAP, there is no consensus on an sPAP cutoff to identify a “hypertensive response”. This led to different definitions of what was a positive test in almost all studies. Some assessed the proportion of patients who achieved a predetermined exercise sPAP over a cutoff (mostly in the 40–50 mmHg range), whereas others analysed the variation/increase in sPAP during exercise. RAP estimation also varied among studies, adding more variability. These variable definitions are important limitations of exercise echocardiography.

Additionally, for a result be considered positive, it should be correlated with a clinical outcome, such as faster progression to resting PAH. However, there are few data on the natural history of EIPH. Two RHC-based studies, with a total of 66 patients with pre-capillary EIPH, observed a PAH incidence of 8–19 % after 2 years [47, 48]. Two recent echocardiographic-based studies are available. A 3.5-year follow up of 170 patients from the cohort of D’Alto et al. identified 6 patients (3.5 %) with incident resting PH [49]. Of these, three developed group-2 PH, one developed group-3 PH and three developed PAH. Among the three patients with PAH, only one had an increase in sPAP >18 mmHg during the index exercise echocardiogram. Recently, another study followed up 40 patients with SSc who had previously undergone exercise echocardiography [50]. After 2 years, 28 % developed resting PH, all belonging to the group of patients with EIPH at the index evaluation. In this cohort, the development of resting PH was associated with factors suggestive of latent LV diastolic dysfunction. The large variation in PH incidence might be related to the absence of confirmation of resting PH by RHC in this latter study. In summary, any variable used to define a positive exercise echocardiographic test has to (1) accurately reflect the pulmonary vascular reserve, which may be achieved using multipoint ΔPAP/ΔCO slopes instead of an isolated sPAP cutoff [51]; (2) integrate information on diastolic function markers, which may be accomplished using a multi-parametric approach for the estimation of LV filling pressures, using E/e’ but also other imaging targets, such as mitral propagation velocity (Vp), diastolic times, pulmonary vein flow measurements or twist analysis [35]; and (3) to be related to outcomes [52]. The ΔPAP/ΔCO slope method that is associated with a comprehensive diastolic function assessment is probably the best suited to comply with all those requirements.

### Other factors to consider

There are also other factors that may influence the results, such as differences in age, gender, athletic capability or levels of adrenergic response among patients [8, 17, 53, 54]. Interstitial lung disease and persistent hypoxia and/or systemic hypertension may also interfere with exercise-induced sPAP [4, 17, 22, 23]. Usually, DL_CO_ is lower in patients with SSc-associated PAH that than in those with idiopathic PAH [55]. This marker, therefore, is considered a valuable predictor of future PAH in SSc, decreasing for 10–15 years before a diagnosis of PAH is made and reaching a mean of 35 % of the predicted value at the time of diagnosis [56–58]. Two authors reported association between lower DL_CO_ and greater risk of developing exercise-induced elevation in estimated sPAP [19, 27]. Although this finding may suggest a common pathophysiological mechanism for both DL_CO_ impairment and exercise PH, further longitudinal studies need to be performed to clarify this relationship.

Lung fibrosis is also associated with a pulmonary hypertensive response to exertion. In the study by D’Alto et al. patients with moderate interstitial lung disease had higher estimated sPAP on exertion than patients without lung disease (39.7 ± 9.3 vs. 36.0 ± 8.4 mm Hg, *p* = 0.016). Once more, several mechanisms can contribute to the elevation of pulmonary pressures during exercise and it is difficult to clearly quantify the contribution of each one of them alone.

### Limitations

As with most systematic reviews, our study is limited by publication bias that may have supported the utility of exercise echocardiography for this indication. We tried to minimize the risk of bias by conducting an extensive search for potentially relevant studies and reviewing all bibliographic references.

### Future perspectives

There is increasing recognition that EIPH may represent a preclinical sign of resting PAH. The standardization of exercise echocardiography for this indication is underway, with several multi-centric initiatives promoting development of a simple and reliable protocol. Concurrently, prospective studies are progressively validating the concept that abnormal responses to exercise, measured with the integration of pressure and flow variables, are associated with a higher incidence of PAH. These studies will provide data to define diagnostic cutoffs that will stratify risk in individual patients and indicate which mechanisms of EIPH (reduced pulmonary reserve or diastolic dysfunction) are the most important in each subject, therefore guiding targeted treatments. Ideally, exercise should be performed in a semi-supine cycloergometer using a ramp protocol, aiming to measure pressure-flow relationships during exertion. Further research will then focus on prophylactic therapeutic interventions aiming to reduce the incidence of PAH in patients at risk.

## Conclusions

In summary, although exercise echocardiography has a strong rationale in the setting of patients with SSc, we found relevant heterogeneity in the methods, the protocols, the expected response to yield a positive result, and critically, no robust data on the prognostic validation of the EIPH concept. Also, the mechanisms whereby pulmonary pressures increase during exercise (either due to PVD, LV diastolic dysfunction, lung disease or a high CO) must be clearly defined and quantified for each patient before the results are to be translated into clinical practice. Current research is addressing those issues to provide a safe, non-invasive tool for preclinical screening of pulmonary vascular disease in patients with systemic sclerosis in the near future.

## Abbreviations

CI, cardiac index; CO, cardiac output; DL_CO_, diffusion capacity of carbon monoxide; E/A, ratio of early diastolic (E) and late diastolic (A) transvalvular velocities; E/e’, ratio of early (e’) diastolic mitral annular velocity to early diastolic (E) wave; EIPH, exercise-induced pulmonary hypertension; FEV1, forced expiratory volume in the first second; FVC, forced vital capacity; HR, heart rate; LV, left ventricle; LVEDP, left ventricular end-diastolic pressure; MET, metabolic equivalent of task; mPAP, mean pulmonary arterial pressure; PAH, pulmonary arterial hypertension; PH, pulmonary hypertension; PVD, pulmonary vascular disease; PVR, pulmonary vascular resistance; RAP, right atrial pressure; sPAP, systolic pulmonary arterial pressure; SSc, systemic sclerosis; TLC, total lung capacity; TPR, total pulmonary resistance

## Additional files

Additional file 1: Table S1. Feasibility, gold-standards and variability assessment. (DOCX 14 kb) Additional file 2: Table S2. Effective Public Health Practice Project Grades classification. (DOCX 12 kb) Additional file 3: Table S3. Exercise echocardiography characteristics. (DOCX 13 kb) Additional file 4: Table S4. Clinical and echocardiographic parameters. (DOCX 14 kb) Additional file 5: Figure S1. Mean pulmonary arterial systolic pressure at rest and on exercise by exercise method/exercise position. (TIFF 77 kb)
