# Supplementary material for: Exercise echocardiography for the assessment of pulmonary hypertension in systemic sclerosis: a systematic review
Source: Arthritis Res Ther. 2016 Jul 2;18:153. doi: 10.1186/s13075-016-1051-9 (PMC4930605; doi:10.1186/s13075-016-1051-9)
Supplement: Additional file 1: Table S1. — Feasibility, gold-standards and variability assessment. (DOCX 14 kb) [file 13075_2016_1051_MOESM1_ESM.docx]

**Additional file 1: Table S1.** Feasibility, gold-standards and variability assessment

| **First author** | **Patients considered for study** | **Patients feasible for echocardiography, n (%)** | **RHC as gold standard** | **Intra and inter-observer variability** |
| --- | --- | --- | --- | --- |
| Mininni | 9 | 9 (100%) | No | No |
| Alkotob | 65 | 65 (100%) | No | No |
| Collins (a) | 9 | 9 (100%) | No | No |
| Collins (b) | 10 | 10 (100%) | No | No |
| Pignone | 27 | 27 (100%) | No | No |
| Huez | 25 | 8 (32%) | No | No |
| Callejas-Rubio | 48 | 41(85%) | No | No |
| Steen | 63 | 54 (86%) | Catheterisation performed in all patients with rest sPAP > 40 mmHg or ≥ 20 mmHg increase during exercise | No |
| Reichenberger | 68 | 33 (49%) | Catheterisation performed in three patients with hypertensive pulmonary vascular response, defined by sPAP increased above 40 mm Hg during cardiopulmonary exercise test or after 2 h hypoxia exposure, respectively | No |
| D’Alto | 212 | 172 (81%) | No | All data were analysed off-line by two observers blinded to the patient conditions. Intra-observer and inter-observer variability was less than 4.0% for measurements at rest and less than 6.0% at maximum exercise. |
| Ciurzynski | 71 | 67 (94%) | Catheterisation performed in all patients with rest sPAP > 31 mmHg or > 20 mmHg increase during exercise | No |
| Baptista | 38 | 23 (61%) | No | No |
| Gargani | 220 | 164 (75%) | No | No |
| Voilliot | 68 | 45 (66%) | No | No |
| Suzuki | 568 | 494 (87%) | No | All data were analysed ofﬂine by two observers blinded to the patient conditions. Intra-observer and inter-observer variability was less than 4.0% for measurements at rest and less than 6.0% after exercise |
| Nagel | 21 | 21 (100%) | Yes | No |
| (a) Systemic sclerosis (b) limited sclerosis  Abbreviations: RHC – right heart catheterisation; sPAP – systolic pulmonary arterial pressure | | | | |
