# Supplementary material for: Exercise echocardiography for the assessment of pulmonary hypertension in systemic sclerosis: a systematic review
Source: Arthritis Res Ther. 2016 Jul 2;18:153. doi: 10.1186/s13075-016-1051-9 (PMC4930605; doi:10.1186/s13075-016-1051-9)
Supplement: Additional file 2: Table S2. — Effective Public Health Practice Project Grades classification. (DOCX 12 kb) [file 13075_2016_1051_MOESM2_ESM.docx]

**Additional file 2: Table S2.** Effective Public Health Practice Project Grades classification

| **First author** | **Selection** | | **Design** | **Confounders** | **Blinding** | | **Data Collection** | **Global Rating** |
| --- | --- | --- | --- | --- | --- | --- | --- | --- |
| Mininni | | Strong | Moderate | Weak | | Weak | Moderate | Weak |
| Alkotob | | Strong | Moderate | Moderate | | Weak | Moderate | Moderate |
| Collins | | Strong | Moderate | Weak | | Moderate | Moderate | Moderate |
| Pignone | | Strong | Moderate | Weak | | Moderate | Moderate | Moderate |
| Huez | | Moderate | Moderate | Weak | | Moderate | Moderate | Moderate |
| Callejas-Rubio | | Strong | Moderate | Weak | | Weak | Moderate | Weak |
| Steen | | Strong | Moderate | Weak | | Weak | Strong | Weak |
| Reichenberger | | Moderate | Moderate | Weak | | Weak | Moderate | Weak |
| D’Alto | | Strong | Moderate | Moderate | | Moderate | Moderate | Moderate |
| Ciurzynski | | Strong | Moderate | Weak | | Moderate | Moderate | Moderate |
| Baptista | | Strong | Moderate | Weak | | Moderate | Moderate | Moderate |
| Gargani | | Strong | Moderate | Strong | | Moderate | Moderate | Strong |
| Voilliot | | Strong | Moderate | Strong | | Moderate | Moderate | Strong |
| Suzuki | | Strong | Moderate | Moderate | | Moderate | Moderate | Strong |
| Nagel | | Strong | Strong | Strong | | Moderate | Strong | Strong |
