# Supplementary material for: Exercise echocardiography for the assessment of pulmonary hypertension in systemic sclerosis: a systematic review
Source: Arthritis Res Ther. 2016 Jul 2;18:153. doi: 10.1186/s13075-016-1051-9 (PMC4930605; doi:10.1186/s13075-016-1051-9)
Supplement: Additional file 3: Table S3. — Exercise echocardiography characteristics. (DOCX 13 kb) [file 13075_2016_1051_MOESM3_ESM.docx]

**Additional file 3: Table S3.** Exercise echocardiography characteristics

| **First author** | **Position** | **Exercise method** | **Workload levels** | **Time point of exercise**  **sPAP measurement** | **Rest HR (bpm)** | **Maximum exercise HR (bpm)** |
| --- | --- | --- | --- | --- | --- | --- |
| Mininni | Supine | Cycloergometer | 140 Watts | During exercise | --- | --- |
| Alkotob | Upright | Treadmill | 69% HR over 85% | After exercise | --- | --- |
| Collins | Upright | Treadmill | MET 5.2 | After exercise | --- | --- |
| Pignone | Supine | Cycloergometer | 86 Watts | During exercise | 81 | 147 |
| Huez | Supine | Cycloergometer | 71 Watts | During exercise | 76 | 126 |
| Callejas-Rubio | Supine | Cycloergometer | 100% HR over 85% | After exercise | --- | --- |
| Steen | Upright | Treadmill | MET 8.1 | After exercise | 81 | --- |
| Reichenberger | Semi-supine | Cycloergometer | 97 Watts | During exercise | --- | 139 |
| D’Alto | Supine | Cycloergometer | 82 Watts | After exercise | 78 | 130 |
| Ciurzynski | Supine | Treadmill | MET 8.3 | After exercise | 82 | 150 |
| Baptista | Semi-supine | Cycloergometer | 64 Watts | During exercise | 71 | 115 |
| Gargani | Supine | Cycloergometer | 76 Watts | During exercise | --- | 138/129* |
| Voilliot | Semi-supine | Cycloergometer | 74 Watts | During exercise | 73 | 118 |
| Suzuki | Supine | Master two-step | --- | After exercise | 73 | --- |
| Nagel | Semi-supine | Cycloergometer | -- | During exercise | --- | --- |

Quantitative results (rest and exercise heart rate) presented by means.

* Exercise-induced heart rate was presented by rate by patients with exercise-induced sPAP<50 mmHg (138 bpm) and patients with exercise-induced sPAP ≥50 mmHg (129 bpm).

Abbreviations: HR – heart rate; MET – metabolic equivalent of task; sPAP– systolic pulmonary arterial pressure**.**
