# Supplementary material for: Exercise echocardiography for the assessment of pulmonary hypertension in systemic sclerosis: a systematic review
Source: Arthritis Res Ther. 2016 Jul 2;18:153. doi: 10.1186/s13075-016-1051-9 (PMC4930605; doi:10.1186/s13075-016-1051-9)
Supplement: Additional file 4: Table S4. — Clinical and echocardiographic parameters. (DOCX 14 kb) [file 13075_2016_1051_MOESM4_ESM.docx]

**Additional file 4: Table S4.** Clinical and echocardiographic parameters

| **First author** | **Exercise-induced**  **sPAP cut-off for a “positive” result** | **Proportion of “positive” tests** | **DL_CO_ (%)** | **Spirometry** | | **Estimated right atrial pressure (mm Hg)*** |
| --- | --- | --- | --- | --- | --- | --- |
|  |  |  |  | **FVC (%)** | **FEV_1_ (%)** |  |
| Mininni | --- | --- | --- | --- | --- | 5; 15 |
| Alkotob | ≥ 40 mm Hg increase | 46% | 59 | 85 | 99 | 5 |
| Collins (a) | ≥ 35 mm Hg increase | 67% | 75 | --- | --- | 10 |
| Collins (b) | ≥ 35 mm Hg increase | 40% | 70 | --- | --- | 10 |
| Pignone | > 40 mm Hg exercise-induced | 67% | 87 | 109 | 109 | --- |
| Huez | --- | --- | --- | --- | --- | --- |
| Callejas-Rubio | --- | --- | 90 | --- | --- | 5 |
| Steen | ≥ 20 mmHg increase | 40% | 63 | 87 | --- | 10 |
| Reichenberger | sPAP > 40 mmHg exercise-induced or after 2 hour hypoxia exposure | 48% | 85 | --- | 91 | --- |
| D’Alto | ≥ 48 mm Hg exercise-induced** | 12%** | --- | --- | --- | 5 |
| Ciurzynski | > 20 mm Hg increase | 16% | 71 | --- | --- | --- |
| Baptista | ≥ 50 mm Hg exercise-induced | 48% | --- | --- | --- | 5 |
| Gargani | ≥ 50 mm Hg exercise-induced | 42% | 70 | --- | 102 | --- |
| Voilliot | > 50 mm Hg exercise-induced | 47% | 65 | 101 | 95 | 10 |
| Suzuki | ≥ 50 mm Hg exercise-induced | 27% | --- | 94 | --- | --- |
| Nagel | ≥ 45 mm Hg exercise-induced | --- | 56 | 96 | 108 | 5;10 |

Quantitative results (DLCO, FVC, FEV_1_, estimated atrial pressure) presented by means.

Results published by Collins *et al* are divided in: (a) patients with diffuse systemic sclerosis and (b) patients with limited systemic sclerosis.

* For eight studies, right arterial pressure (RAP) was assumed, instead of estimated by echocardiographic findings. Four studies assumed a RAP of 5 mm Hg and three assumed 10 mm Hg. Minini *et al.* assumed a RAP of 5 mmHg was assumed when inferior vena cava diameter decreased more than 50% after deep inspiration and 15 mmHg if less than 50%. Nagel et al. assumed a RAP of 5 mmHg was assumed when inferior vena cava diameter was less than 20 mm and decreased after deep inspiration and 10 mmHg if it was at least 20 mm.

** Data not obtained directly from the article published by D’Alto *et al* (D'Alto *et al*. Heart 2011;97:112-7), but instead from Codullo *et al*. (Codullo V, et al. Arthritis and rheumatism 2013;65:2403-11), which followed the same cohort.

Abbreviations: AP – atrial pressure; DLCO – diffusing capacity of the lung for carbon monoxide (% of predicted); FEV_1_ – forced expired volume in 1 second (% of predicted); FVC – forced vital capacity (% of predicted); HR – heart rate; sPAP – systolic pulmonary arterial pressure.
