# Supplementary figures and images for: Exercise echocardiography for the assessment of pulmonary hypertension in systemic sclerosis: a systematic review
Source: Arthritis Res Ther. 2016 Jul 2;18:153. doi: 10.1186/s13075-016-1051-9 (PMC4930605; doi:10.1186/s13075-016-1051-9)

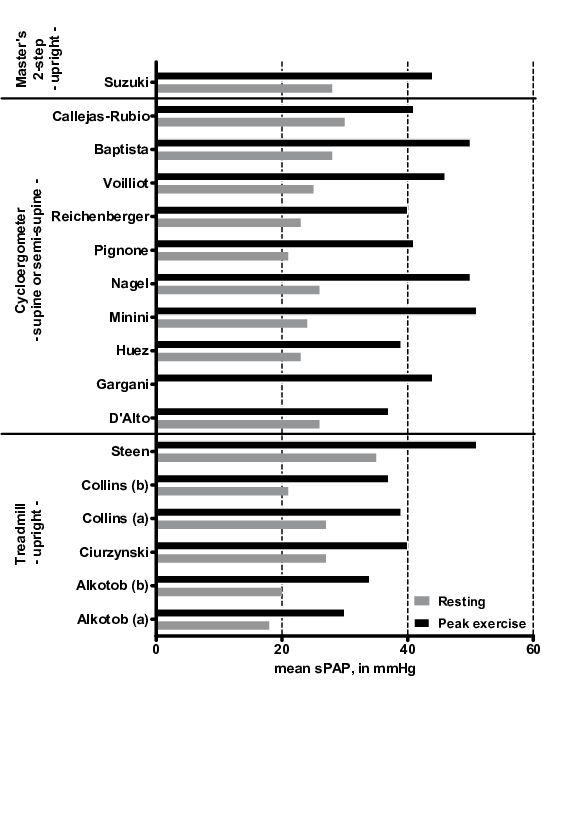

Supplement: Additional file 5: Figure S1. — Mean pulmonary arterial systolic pressure at rest and on exercise by exercise method/exercise position. (TIFF 77 kb) [file 13075_2016_1051_MOESM5_ESM.tiff]
